# Complete chloroplast genome sequence of the rose apple, *Syzygium jambos* (Myrtaceae)

**DOI:** 10.1080/23802359.2020.1826000

**Published:** 2020-10-05

**Authors:** Zhixia Sun, Qingping Huang, Chen Feng

**Affiliations:** a Fujian Provincial Key Laboratory of the Development and Utilization of Bamboo Resources, Sanming University, Sanming, China; b Key Laboratory of Plant Resources Conservation and Sustainable Utilization, South China Botanical Garden, Chinese Academy of Sciences, Guangzhou, China

**Keywords:** *Syzygium*, chloroplast genome, Illumina sequencing, traditional medicine

## Abstract

Plants in the genus *Syzygium* have been widely used as traditional medicine, fruit crops, and ornamental trees. In this study, we reported the complete chloroplast genome of *Syzygium jambos* (L.) Alston which was known as rose apple. The chloroplast genome of this species is 158541 bp in length, including a pair of inverted repeat regions (IRs) (26076 bp) that is divided by a large single copy area (LSC) (88036 bp) and a small single copy area (SSC) (18353 bp). The circular chloroplast genome of *S. jambos* contains 132 unique genes, composing of 85 protein-coding genes, 39 tRNA genes and 8 rRNA genes. Phylogenetic analysis indicates that *S. jambos* is clustered with species in genus *Syzygium*. This complete chloroplast genome of *S. jambos* will provide a powerful tool to accelerate breeding, biotechnological and phylogenetic study.

*Syzygium* Gaertn. is the largest genus in Myrtaceae, comprises about 1200 species and is distributed in the Old World tropics and subtropics (Biffin et al. 2010; Soh and Parnell 2015). Some species of genus *Syzygium* have been widely used as fruit crops (e.g. *S. samarangense*; Hao et al. 2016), ornamental trees (e.g. *S. cumini*; Abreu-Harbich et al. 2015) and traditional herbal medicines (e.g. *S. aromaticum*; El-Shouny et al. 2020). *Syzygium jambos* (L.) Alston, which is known as rose apple, is one of the most important species in *Syzygium*. It is native to southeast Asia and is cultivated in some areas of the tropics (Lim 2012). *S. jambos* has been reported as medicines to treat diabetes, inflammation and gastrointestinal disorders (Murugan et al. 2011; Rezende et al. 2013) for its leaf and fruit extracts containing high concentrations of tannins, phenolic acids, and other antioxidants (Oliveira et al. 2005; Gavillán-Suárez et al. 2015). Edible fruits of this species have a high percentage of pulp and contain an attractive aroma (roselike odour) and taste (sweet and slightly acidic), and are often used to make juices, jellies and jams (Guedes et al. 2004). In addition, *S. jambos* is also planted as ornamental trees in many countries for its outstanding adaptability. Most studies of *S. jambos* have focused on its chemical extraction and pharmacological properties. However, few genomic resources have been reported in this species, except a few gene sequences in phylogenetic analysis (Biffin et al. 2006).

Chloroplast genes and conserved sequences are often utilized for phylogenetic analysis and domestication studies of higher plants (Jansen et al. 2007). The whole chloroplast genome sequences have also been demonstrated the potential to understand structure and functional evolution (Jansen et al. 2007; Moore et al. 2010). In genus *Syzygium*, the chloroplast genome of some species such as *S. samarangense* and *S. forrestii* has been reported (Liu et al. 2018; Zhang et al. 2019), but the chloroplast genome of *S. jambos* has not been reported. Here, we sequenced and analyzed the complete chloroplast genome sequence of *S. jambos* based on the Illumina sequencing data. This study aimed to characterize the complete chloroplast genome sequence of *S. jambos* as a resource for future genetic studies.

Fresh leaves of *S. jambos* were collected from South China Botanical Garden, Chinese Academy of Sciences (Guangzhou, China). Total genomic DNA was extracted for library construction and sequencing. Voucher specimens of *S. jambos* were deposited at the herbarium of South China Botanical Garden (accession number: SCBG-CF-2061). The library was constructed with the insertion size of 350 bp. The high-throughput sequencing (pair-end 150 bp) was performed on an Illumina XTen platform. The clean reads were assembled by using the program NOVOPlasty (Dierckxsens et al. 2017). A ribulose-1, 5-bisphosphate carboxylase/oxygenase (*rbc*L) gene sequence from *S. samarangense* (GenBank accession: MH371141) was used as seed sequence, and the whole chloroplast genome sequences of *S. samarangense* and *S. forrestii* (MK102721) were used as a reference to resolve the inverted repeat in the chloroplast genome of *S. jambos*. The assembled chloroplast genome was annotated by the combination of PGA (Qu et al. 2019) and GeSeq (Tillich et al. 2017). For necessary genes, we manually corrected their positions of start and stop codons and boundaries between exons and introns. The annotated chloroplast genomic sequence has been deposited in GenBank with an accession number: MT731620.

The complete chloroplast genome of *S. jambos* is 158,541 bp in length, and has a typical quadripartite construction, which contains two inverted repeat regions (IRa and IRb) of 26,076 bp that is insulated by a large single-copy (LSC, 88,036 bp) and a small single-copy (SSC, 18,353 bp). The total GC content of complete chloroplast genome, LSC, SSC, IR regions is 37.0%, 55.5%, 11.6% and 32.9%, respectively. The complete chloroplast genome of *S. jambos* contains 132 unique genes, including 85 protein-coding genes, 39 tRNA genes and 8 rRNA genes. Introns are present in 18 of the annotated genes. Three of the intron containing genes (clpP, rps12, and ycf3) contain three exons. Most of these genes are single-copy genes. However, 18 genes were duplicated in IR regions.

To confirm the phylogenetic position of *S. jambos*, the complete chloroplast genomes of 15 published species within Myrtaceae and one outgroup (*Punica granatum*, Lythraceae, MK603512) were downloaded from the NCBI GenBank database. Ninety-four chloroplast genes shared by all species we analyzed were extracted, and were aligned by using MUSCLE (Edgar 2004). We concatenated these genes and then constructed a maximum likelihood tree (Figure 1) using IQ-TREE (Nguyen et al. 2015). Phylogenetic analysis strongly supported that *S. jambos* was closely related to species in genus *Syzygium* (Figure 1), which is consistent with the previous studies in Myrtaceae (Biffin et al. 2010; Thornhill et al. 2015). In conclusion, this published *S. jambos* chloroplast genome will provide a solid foundation for phylogenetic and evolutionary studies in *Syzygium* and is expected to improving the understanding of molecular mechanisms under pharmacological properties of *S. jambos*.

**Figure 1.**
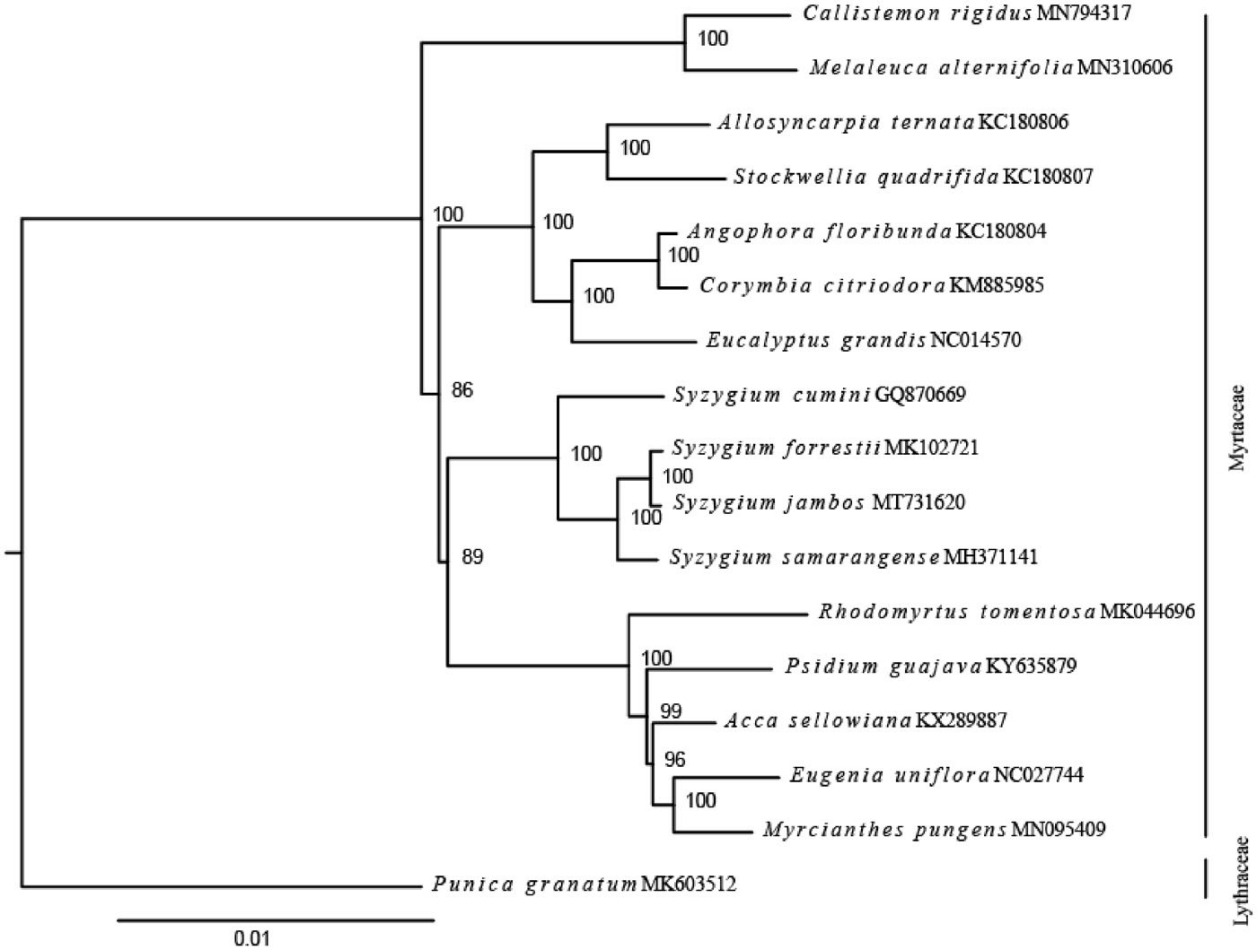
Maximum-likelihood tree shown the relationship among *S. jambos* and other 16 species within Myrtaceae and one outgroup species (*Punica granatum*), using chloroplast gene sequences. Bootstrap supports based on 1000 replicates are given at the node.

## Data Availability

The raw sequencing data of *S. jambos* have been deposited in the NCBI Sequence Read Archive under accession numbers PRJNA658704. The chloroplast genome of the *S. jambos* was submitted to GenBank under accession number: MT731620. Treefile of 18 species and genes for phylogenetic analysis were deposited at Figshare: https://doi.org/10.6084/m9.figshare.12818804.v2.
